# Impact of Co-Administration of N-Acetylcysteine and Vitamin E on Cyclophosphamide-Induced Ovarian Toxicity in Female Rats

**DOI:** 10.1155/2022/9073405

**Published:** 2022-08-23

**Authors:** Mahdieh Raeeszadeh, Seed Mohammad Saleh Hosseini, Ali Akbar Amiri

**Affiliations:** ^1^Department of Basic Sciences, Sanandaj Branch, Islamic Azad University, Sanandaj, Iran; ^2^Graduate of Faculty of Veterinary Sciences, Sanandaj Branch, Islamic Azad University, Sanandaj, Iran

## Abstract

Cyclophosphamide is used to treat various types of cancer. However, it can reduce ovarian function and fertility rate. The current study was done to compare the effects of N-acetylcysteine and vitamin E on cyclophosphamide-induced ovarian damage. Thirty-five rats were randomly divided into 5 groups: control (C), cyclophosphamide (CP, 200 mg/kg single dose intraperitoneally), T1 (cyclophosphamide + vitamin E at 200 mg/kg), T2 (cyclophosphamide + 200 mg/kg N-acetylcysteine), and T3 (cyclophosphamide + N-acetylcysteine and vitamin E at 200 mg/kg). The main measurements included total antioxidant capacity (TAC), glutathione peroxidase (GPx), malondialdehyde (MDA), interleukin 8 (IL-8), tumor necrosis factor-*α* (TNF*α*), follicle stimulating hormone (FSH), luteinizing hormone (LH), and estrogen (ES). Except for the C and T3 groups, the other groups lost weight. A significantly lower concentration of MDA was observed in the T3 group. However, TAC was substantially increased compared to the other groups. The level of GPx in the S group was significantly reduced compared to all groups. Proinflammatory markers (IL-8 and TNF*α*) reached their lowest serum level in the T3 group, with a statistically significant difference compared to that of the S group. In addition, there were no significant differences in the means of primary, secondary, and graph and atretic follicles between the T3 and C group. On the other hand, a decrease in FSH and LH was observed while an increase in ES was seen in the T3 group compared to the S group. This study revealed that N-acetylcysteine and vitamin E coadministration could significantly decrease the side effects of cyclophosphamide, especially in ovarian tissue.

## 1. Introduction

There are different conventional treatments for cancer including, surgery, radiotherapy, and drug use [1, 2]. Cyclophosphamide (CP) is a cytotoxic anticancer agent used as chemotherapy to suppress the immune system but has some adverse effects, especially on the ovaries. The most crucial function of the ovary is the production of female gametes or egg cells/ovum. The mammals' ovum multiplies only during the embryonic period. After birth, there is a limited number of follicles stored in the ovary, and depletion of these follicles leads to preterm aging of the ovary [3].

Cancer treatment using CP in female patients has been shown to cause ovarian dysfunction. CP and other alkylating agents may cause gene mutations, chromosomal breakdowns, and aneuploidy in somatic cells [4]. CP also destroys primary follicles and damages the reproductive system due to reduced follicular storage. As a result, it leads to infertility and early menopause in women [5].

A key mechanism of CP treatment is to increase the production of oxygen-free radicals such as reactive oxygen species (ROS). When the production of free radicals is faster than their neutralization by antioxidant mechanisms, oxidative stress occurs [6, 7]. CP also induces inflammation [8], as an immune response to infection, tissue damage, or chemical toxicity, and involves the expression of cytokines as immunomodulatory agents [9]. Research has shown that increased expression of cytokines in cells can enhance programmed cell death by activating IFN-*α* and IFN-*γ* [10].

Antioxidants prevent cell damage by reacting and scavenging oxidizing free radicals. Therefore, they are helpful in chemotherapy. As the recent statistics reveal, 13 to 87% of patients with cancer use antioxidant supplements [11]. N-acetylcysteine (NAC) is a commonly used antioxidant that is highly effective, although its ROS reactivity is limited. NAC can directly eliminate reactive oxygen species to protect cells from oxidative damage and can stimulate glutathione synthesis. Moreover, NAC modulates oxidative stress, liver damage, and inflammation of different organs [12]. It is reported that N-acetylcysteine may have some beneficial effects on gonadotoxicity and genotoxicity after using CP in animals [13].

It is important to find the most efficient compounds for reducing the side effects of CP on ovaries and female infertility. The enzymatic antioxidants such as vitamins E and C have the potential to block cascade reactions created by free radicals through hydrogenating free-radical molecules [14]. However, antioxidants follow different mechanisms in protecting organs from oxidative damage and exerting antitumor effects [15].

Therefore, this study aimed to evaluate the modulating performance of NAC and vitamin E on the effects of CP in the ovaries of female rats.

## 2. Methods

The present study was an experimental study performed on 35 female Wistar rats (weight ranged 200-230 g and age ranged 6-8 weeks) obtained from Pasteur Institute of Iran.

All animals in the study had free access to safe water and chow (Behparvar Company, Tehran, Iran) and were preserved under controlled environmental conditions (temperature 20-21°C, 12 hours of light every 24 hours). The study was started seven days after the animals adapted to the environmental conditions. In this study, animals were also cycled by estradiol valerate (Iran Hormone-Iran Company) and progesterone (Iran Hormone-Iran Company) to prepare tissue samples using a vaginal smear.

### 2.1. Preparation of Chemical Compounds

Pure vitamin E powder (CAS number: 7695-91-2) with a molecular weight of 472.70 g/mol and N-acetylcysteine (CAS number: 616-91-1) with a molecular weight of 163.193 g/mol were purchased from a life science company (Sigma-Aldrich, USA). Cyclophosphamide was also purchased from the same company (CAS number: 605-19-2) with a molecular weight of 279.10 g/mol and a purity of more than 99.5%.

### 2.2. Study Design

Cycled female Wistar rats were randomly divided into five groups of 7 as follows:

- Group (C): rats were given only distilled water and chow
- Group (S): rats were given cyclophosphamide single i.p. injections of CP (200 mg/kg of body weight) [16]
- Group (T1): rats received cyclophosphamide and vitamin E at a dose of 200 mg/kg daily gavage
- Group (T2): rats received cyclophosphamide and n-acetylcysteine at a dose of 200 mg/kg daily gavage
- Group (T3): rats received cyclophosphamide with n-acetylcysteine and vitamin E [17]

The study period was 21 days. At the end of the study, the animals were weighed. A vaginal smear was used to confirm estrous cycle synchrony. Then, samples were fixed in ethanol (96%) and switched by hematoxylin and eosin. The animals in the estrous phase (horn cells without nuclei) were euthanized by microscopic examination [18]. They were anesthetized by intraperitoneal injection of ketamine (100 mg/kg) and xylazine (10 mg/kg). Blood samples were taken from the hearts of animals under anesthesia, and serum samples were used to measure total antioxidant capacity (TAC) using the FRAP method. Also, hormonal assessment and proinflammatory cytokines measurements were performed using the ELIZA method. Glutathione peroxidase (GPx) and malondialdehyde (MDA) were measured in right ovarian tissue by a ZELLBIO calorimetric kit [19]. For histopathological examination, the left ovary was fixed in 10% formalin buffer after separation.

In this study, ethical considerations for the use of animals were based on international protocols [20]. They were confirmed by the ethics committee of the Kurdistan University of Medical Sciences with the code IR. MUK.REC.1398.58.

### 2.3. Measurement of TAC Level Using FRAP Method

In this method, the ability of plasma to reduce ferric ions is measured. At acidic pH, when the Fe III-TPTZ complex was reduced to Fe II, the blue color was produced, which is read at 593 nm. TAC values were measured using a standard diagram with a concentration of 100-1000 *µ*mol/L [21].

### 2.4. Assessment of GPx Level in Ovarian Tissue

Using a commercial kit (ZELLBIO Co., Germany) with the number ZB-GPX-A11, the GPx level in the ovary was measured using the colorimetric method. Ten *μ*l of homogenized ovarian tissue with 200 *μ*l of prepared reagent was mixed, and after 5 minutes of incubation at room temperature, the GPx concentration was measured at 412 nm [22].

### 2.5. Measurement of MDA Concentration in Ovarian Tissue

To homogenate 500 *μ*l of ovarian tissue, 1.5 ml of 10% trichloroacetic acid was added and centrifuged for 10 minutes. Then, 1.5 ml of the supernatant was removed, and 2 ml of 0.067% thiobarbituric acid was added and boiled for 30 minutes. Then, the absorption of pink dye solution on the wavelength of 532 nm was read by a spectrophotometer [23].

### 2.6. Analysis of Serum Proinflammatory Cytokines

The serum levels of IL-8 interleukin and TNF*α* were measured using enzyme-linked immunosorbent assay kits (ELISA) (USCN, Wuhan, China) based on constructor protocols at 450 nm. Then, the results were expressed in pg/mL [24].

### 2.7. Evaluation of the Histological Ovary

For histological evaluation of the prepared tissue, at least five sections of each ovarian section were examined with a light microscope (Nikon 3200), and primary, secondary, graph, corpus luteum, and atretic follicles in ovarian tissue were evaluated.

### 2.8. Measurement of Hormones

Enzyme-linked immunosorbent assay (ELISA) kits (USCN, Wuhan, China, Cat N: 532.40) were used to measure the FSH, LH, and E2 levels according to the manufacturer's protocols (microplate reader, Stat Fax 4200).

### 2.9. Data Analysis

The study results were reported as means ± standard errors (mean ± SEM). To compare the mean values of parametric data between the groups, a one-way ANOVA and Tukey post-hoc test were performed. All statistical analyses of the data were administered using SPSS 23 software, and the significance level was set *P* < 0.05.

## 3. Results

### 3.1. Changes in Animal Weight in the Studied Groups

According to Table 1, the lowest final weight was seen in the S group and the highest in the control group. Significant differences were found in the final weight of animals in the control group and other groups (*P* < 0.01). There was also substantial weight loss in all groups except the control group and T3 group.

### 3.2. MDA Concentration in the Studied Groups

**Figure 1:**
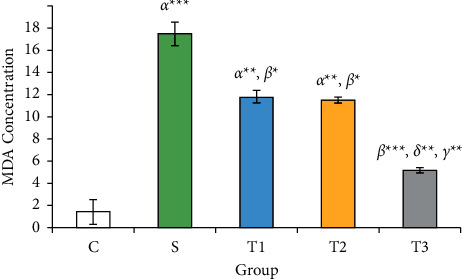
MDA concentration in ovarian tissue of studied animals. *C*: distilled water; *S*: CP; T1: CP + Vit. E; T2: CP + NAC; T3: CP + NAC + Vit. E. Each column represents the mean ± standard error (Mean ± SEM).  ^*∗*^*P* < 0.05,  ^*∗*^ ^*∗*^*P* < 0.01, and  ^*∗*^ ^*∗*^ ^*∗*^*P* < 0.001. *α*, *β*, *γ*, and *δ* are compared with groups *C*, *S*, *T*1, and *T*2, respectively.

Figure 1 indicates the ovarian MDA levels in S groups (17.49 ± 1.05), *T1* (11.79 ± 0.58), and *T*2 (11.48 ± 0.26) ratios: the control group (1.45 ± 1.11) *µ*mol/mg showed a significant increase (*P* < 0.05). Also, there was a significant decrease in ovarian tissue MDA level in group *T*3 (5.18 ± 0.22) *µ*mol/mg compared to the *S* group (*P* < 0.001). However, the reduction of malondialdehyde was not statistically significant between *T*1 and *T*2 groups.

### 3.3. TAC Concentration in Serum of the Studied Groups

**Figure 2:**
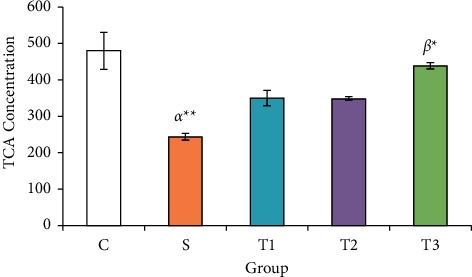
Serum TAC levels of animals in the study groups. Each column represents the mean ± standard error (mean ± SEM). ^*∗*^*P* < 0.05 and  ^*∗*^ ^*∗*^*P* < 0.01. *α* and *β* compared with groups *C* and *S*, respectively.

TAC value in the S group (244.30 ± 9.39 *µ*mol/L) showed a significant difference between the control group (480.32 ± 51.17) and the *T*3 group (437.40 ± 8.83 *µ*mol/L) (*P* < 0.01) (Figure 2).

### 3.4. GPx Levels in the Studied Groups

**Figure 3:**
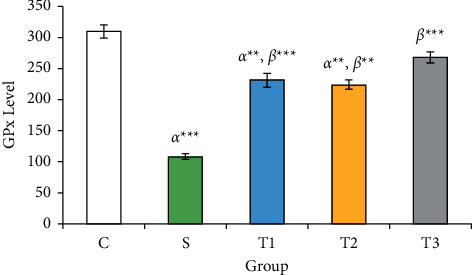
GPx levels of animals in the studied groups. Each column represents the mean ± standard error (Mean ± SEM).  ^*∗*^ ^*∗*^*P* < 0.01 and  ^*∗*^ ^*∗*^ ^*∗*^*P* < 0.001. *α* and *β* compared with groups *C* and *S*, respectively.

GPx level in the *S* group (108.20 ± 4.35 U/ml) compared to the control group (309.42 ± 11.01 U/ml) showed a significant decrease (*P* < 0.001). GPx was significantly increased in *T*1 (230.84 ± 10.95), *T*2 (223.83 ± 7.36), and *T*3 groups (268.13 ± 8.37 U/ml) compared to the *S* group (*P* < 0.05) (Figure 3).

### 3.5. Ovarian Tissue Cytology Results in the Studied Groups

**Figure 4:**
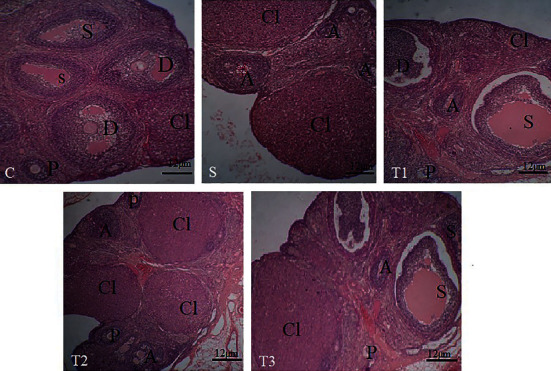
Ovarian microscopic sections in the studied groups. P: primary follicle; S: secondary follicle; D: graph follicle; Cl: corpus luteum; A: atretic follicle. Hematoxylin-eosin staining (magnification 400).

As shown in Table 2, the primary follicles in the S group significantly decreased compared to other groups (*P* < 0.05). The number of secondary follicles in the study groups did not significantly change (*P* > 0.05). Graph follicle count in *S*, *T*1, *T*2, and *T*3 groups showed a significant decrease compared to the *C* group (*P* < 0.05). The corpus luteum (CL) in *T*2 and *T*3 groups had a significant increase compared to the *S* group. Also, the CL increased significantly in the *T*3 group compared to the *T*1 group (*P* < 0.05). The count of atretic follicles in the *T*1 and *T*3 groups showed a significant decrease compared to the *S* group (*P* < 0.05) (Figure 4).

### 3.6. Changes in Proinflammatory Biomarkers in Different Groups

According to Table 3, the highest concentrations of TNF*α* and IL-8 were in the S group, and the lowest were in the control group. In the *T*3 group, there is a significant decrease in proinflammatory cytokines.

### 3.7. Changes in Serum Hormones

Based on Table 4, the S group showed the highest levels of the FSH and LH hormones compared to the other groups, while FSH and LH showed a significant decrease in the T3 group, and E2 showed an increase (*P* < 0.05).

## 4. Discussion

Due to the side effects reported for cyclophosphamide, especially in the ovarian tissue, this interventional study evaluated the effects of vitamin E and N-acetyl in reducing the harmful effects of this compound.

In the current study, we found that the combined use of Vit. E and NAC inhibited the loss of animal body weight, which can be a consequence of a protective effect against cyclophosphamide toxicity. Moreover, this study revealed that under the treatment of co-administration of NAC and Vit. E, MDA concentration in the ovary could be decreased, and the concentration of antioxidant enzymes GPx and TAC increased, showing that the compound has a positive antioxidant effect. In addition, the decrease in proinflammatory biomarkers of IL-8 and TNF*α* in the group (Vit. E + NAC) compared to that of other groups emphasizes the combination of these two antioxidants and anti-inflammatory functions in experimental cyclophosphamide-induced ovarian toxicity. Furthermore, the hormonal results and the microscopic view of the ovarian follicles confirm the establishment of the estrous cycle, the increase of primary and secondary follicles, and the reduction of atretic follicles in the combined Vit. E and NAC.

The decrease in body weight in the group treated with CP observed in this study may be attributed to anorexia which might suggest that CP has harmful effects on the gastrointestinal tract or appetite center in the hypothalamus [25, 26]. Hence, using antioxidants can improve the physical condition and weight gain in animals [27].

Inflammation is a physiological process in ovulation. Nevertheless, uncontrolled inflammation has destructive effects on hormone production and ovulation [28, 29]. In the ovary, ROS also increases during ovulation and steroidogenesis. Therefore, ROS detoxification is highly important in oocyte maturation and fetal development. ROS interferes with ovarian function by inducing lipid peroxidation and MDA production [30].

Lipid peroxidation in the plasma membrane of luteal cells causes damage to gonadotropin receptors and thus decreases steroidogenesis in the corpus luteum [31]. In terms of hormonal status, this study showed a significant decrease in estrogen and progesterone after CP administration, which is consistent with other studies. In his research, Saleh showed that CP causes severe toxicity in the ovary through oxidative stress and inflammation and subsequently decreases the level of E2 and anti-Müllerian hormone (AMH) in the serum [32]. However, the practice of antioxidants by reducing oxidative damage and inflammation induced by cyclophosphamide has a significant effect on the maturation of follicles and positive ovarian function.

In this regard, we can refer to parallel studies. Yener et al. (2013) showed a positive effect of spirulina on cyclophosphamide-induced ovarian toxicity in mice. Melekoglu et al. (2018) also found beneficial effects of curcumin and capsaicin on cyclophosphamide-induced premature ovarian failure in a rat model [33, 34].

As an antioxidant, NAC can synthesize glutathione and help to prevent the accumulation of free radicals [35]. Vitamin E (alpha-tocopherol), as a fat-soluble antioxidant vitamin, also plays an important role in protecting body tissues against oxidative stress. Vitamin E inhibits lipid peroxidation by reacting with hydroxyl radicals in the membrane and also acts as a scavenger of free radicals as well. In line with this result, NAS alignment reduces ovarian ischemia/reperfusion oxidative damage by reducing MDA levels [36]. Moreover, it is possible to refer to the administration of vitamin E to prevent liver tissue damage caused by CP [15]. Kyung Soo Kang et al. announced the hepatoprotective effect of N-acetyl in cyclophosphamide poisoning by reducing IL-8, IL-10, TNF, and interferon [12].

Glutathione (GSH) deficiency is associated with numerous pathological conditions. Administration of NAC, a cysteine prodrug, replenishes intracellular GSH levels [37].

Furthermore, Naglaa Fathi Khedr reported that mirtazapine (MTZ) and hesperidin (HSP) could promote ovarian protection against damage due to CP chemotherapy by increasing SOD, GPx, and decreasing MDA levels [38].

On the other hand, cytokines affect follicle maturation and angiogenesis and indirectly deliver nutrients to the follicle. In the current study, the decrease in the concentration of proinflammatory markers (IL-8 and TNF*α*) in the combination group of NAC and Vit. E compared to cyclophosphamide is the other result of the positive effects of this intervention [39, 40].

Histological observations showed that CP reduces ovarian follicles and increases atretic follicles. The combination of NAC + Vit. E supports the growth of damaged follicles and modulates the immune system, and maintains the oxidative balance. The findings of the present study were thus confirmed [40, 41].

## 5. Conclusion

The results of the study revealed the synergistic effect of Vit. E and NAC, as known antioxidant compounds have strong antioxidant properties, which result in weight gain, decreased MDA levels, and increased levels of TAC and GPx antioxidants in the case of cyclophosphamide. However, IL-8 and TNF*α* decreased to balanced serum levels. These biochemical reactions increased the average ovarian follicles and improved fertility. In addition, the decrease in FSH and LH with the increase in estradiol in reducing the ovarian complications of CP were the other proofs of the effectiveness of NAC and Vit. E.

However, NAC had a better effect than vitamin E in reducing the side effects of CP on the ovary, although their cumulative effect significantly increased the ovarian function in CP poisoning. Evaluation of the function of other parameters of oxidative stress and proinflammatory biomarkers in the ovarian tissue, and its molecular reactions can help advance our results.

## Figures and Tables

**Table 1:** Initial, final weight, and weight changes at the end of the study in groups.

Variable	Group				
	*C*	*S*	*T*1	*T*2	*T*3
Initial body weight (g)	225.46 ± 5.38	228.38 ± 6.46	230.96 ± 7.04	227.60 ± 4.18	231.54 ± 3.12
Final body weight (g)	256.48 ± 5.86	176.70 ± 3.73 *α*^∗∗^	204.01 ± 2.04 *α*^*∗*^, *β*^*∗*^	200.76 ± 3.35 *α*^*∗*^, *β*^*∗*^	234.12 ± 5.27 *β*^∗∗^, *γ*^*∗*^
Weight gain (g)	31.02 *α* ± 0.48	−51.68 ± 2.73*α*^∗∗^	−26.95 ± 5.03*α*^∗∗^, *β*^*∗*^	−26.84 ± 1.24*α*^∗∗^, *β*^*∗*^	2.58 ± 3.18*α*^*∗*^, *β*^*∗*^, *γ*^*∗*^

*C*: distilled water; *S*: CP; *T*1: CP + Vit. E; T2: CP + NAC; T3: CP + NAC + Vit. E. Each raw represents the mean ± standard error (mean ± SEM).  ^*∗*^*P* < 0.05,  ^*∗*^ ^*∗*^*P* < 0.01, and  ^*∗*^ ^*∗*^ ^*∗*^*P* < 0.001. *α*, *β*, *γ*, and *δ* are compared with groups *C*, *S*, *T*1, and *T*2, respectively.

**Table 2:** Changes in animal ovarian cells in the experimental groups.

Ovarian follicles	Group				
	*C*	*S*	*T*1	*T*2	*T*3
Primary follicle	5.53 ± 0.58	3.48 ± 0.62	4.88 ± 0.83	5.16 ± 0.48	5.66 ± 0.86 *β*^*∗*^
Secondary follicle	4.36 ± 0.38	3.86 ± 0.61	2.73 ± 0.73	3.00 ± 0.16	2.85 ± 0.54
Graph follicle	5.43 ± 0.62	1.77 ± 0.43 *α*^∗∗^	2.15 ± 0.79 *α*^∗∗^	1.63 ± 0.58 *α*^∗∗^	3.11 ± 0.23 *α*^*∗*^
Corpus luteum	6.63 ± 0.51	4.49 ± 0.62	5.11 ± 0.23	7.51 ± 0.72 *β*^*∗*^	8.43 ± 0.65 *β*^∗∗^, *γ*^*∗*^
Atretic follicle	4.27 ± 0.38	5.28 ± 0.62	3.00 ± 0.13 *β*^*∗*^	3.59 ± 0.58	3.17 ± 0.78 *β*^*∗*^

All values are presented as mean ± standard error (mean ± SEM). ^*∗*^*P* < 0.05 and  ^*∗*^ ^*∗*^*P* < 0.01. *α*, *β*, and *γ* compared with groups *C*, *S*, and *T*1, respectively.

**Table 3:** Changes in proinflammatory markers in the study groups.

Parameters	Group				
	*C*	*S*	*T*1	*T*2	*T*3
TNF*α* (pg/ml)	156.8 ± 14.56	365.59 ± 27.58 *α*^∗∗^	250.67 ± 22.4 *α*^∗∗^, *β*^∗∗^	204.68 ± 14.80 *α*^*∗*^, *β*^∗∗^	178.28 ± 18.79 *α*^*∗*^, *β*^∗∗^
IL-8 (pg/ml)	170.57 ± 12.34	304.56 ± 11.24 *α*^∗∗^	203.67 ± 8.37 *α*^*∗*^, *β*^∗∗^	200.47 ± 7.90 *α*^*∗*^, *β*^∗∗^	196.69 ± 10.36 *α*^*∗*^, *β*^∗∗^

All values are presented as mean ± standard error (mean ± SEM). ^*∗*^*P* < 0.05 and  ^*∗*^ ^*∗*^*P* < 0.01. *α* and *β* compared with groups *C* and *S*, respectively.

**Table 4:** Mean ± SEM of FSH, LH, and E2 in different groups.

Hormones	Group				
	*C*	*S*	*T*1	*T*2	*T*3
FSH (U/ml)	2.43 ± 1.25	12.31 ± 3.97 *α*^∗∗^	6.67 ± 1.27 *α*^∗∗^, *β*^∗∗^	5.00 ± 1.30 *α*^∗∗^, *β*^∗∗^	3.45 ± 1.08 *β*^∗∗^, *γ*^*∗*^
LH (U/ml)	2.87 ± 0.70	11.50 ± 1.24 *α*^∗∗^	7.85 ± 0.93 *α*^∗∗^, *β*^∗∗^	5.72 ± 1.27 *α*^∗∗^, *β*^∗∗^	4.68 ± 1.20 *α*^*∗*^, *β*^∗∗^, *γ*^*∗*^
E2 (pg/ml)	27.49 ± 4.48	11.18 ± 2.195 *α*^∗∗^	15.23 ± 3.28*α*^∗∗^	15.13 ± 5.57*α*^*∗*^	18.20 ± 4.30*α*^*∗*^, *β*^*∗*^

All values are presented as mean ± standard error (mean ± SEM). ^*∗*^*P* < 0.05 and  ^*∗*^ ^*∗*^*P* < 0.01. *α*, *β*, and *γ* compared with groups *C*, *S*, and *T*1, respectively.

## Data Availability

The data used to support the findings of this study are available from the corresponding author upon request.
